# Unravelling the Mechanistic Understanding of Metal Nanoparticle-Induced Reactive Oxygen Species Formation: Insights from a Cu Nanoparticle Study

**DOI:** 10.1021/acs.chemrestox.3c00177

**Published:** 2023-11-10

**Authors:** Amanda Kessler, Ping Huang, Eva Blomberg, Inger Odnevall

**Affiliations:** † KTH Royal Institute of Technology, Department of Chemistry, Division of Surface and Corrosion Science, SE-100 44 Stockholm, Sweden; ‡ Department of Chemistry − Ångström Laboratory, Uppsala University, Box 523, SE-751 20 Uppsala, Sweden; § AIMES−Center for the Advancement of Integrated Medical and Engineering Sciences at Karolinska Institute and KTH Royal Institute of Technology, SE-100 44 Stockholm, Sweden; ∥ Department of Neuroscience, Karolinska Institute, SE-171 77 Stockholm, Sweden

## Abstract

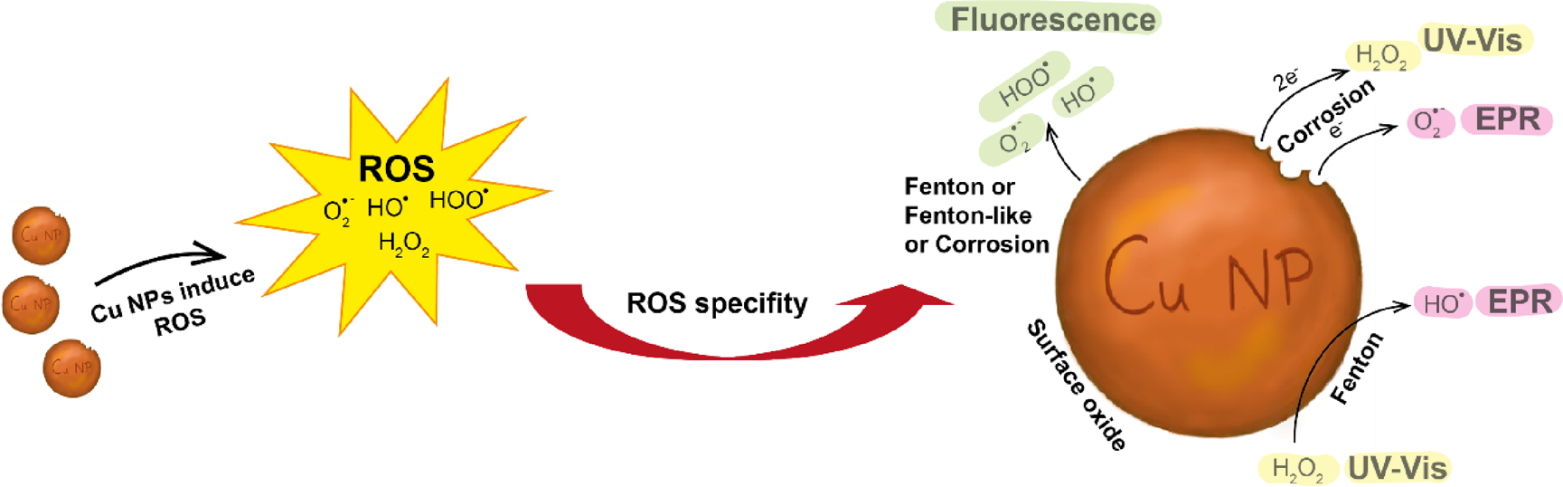

Humans can be exposed to engineered and nonintentionally formed metal and metal oxide nanoparticles (Me NPs) in occupational settings, in public transportation areas, or by means of contact with different consumer products. A critical factor in the toxic potency of Me NPs is their ability to induce oxidative stress. It is thus essential to assess the potential reactive oxygen species (ROS) formation properties of Me NPs. A common way to assess the relative extent of ROS formation *in vitro* is to use fluorescence spectroscopy with the DCFH-DA (2′,7’-dichlorofluorescein diacetate) probe, with and without HRP (horseradish peroxidase). However, this method does not provide any information about specific ROS species or reaction mechanisms. This study investigated the possibility of using complementary techniques to obtain more specific information about formed ROS species, both the type and reaction mechanisms. Cu NPs in PBS (phosphate buffered saline) were chosen as a test system to have the simplest (least interference from other components) aqueous solution with a physiologically relevant pH. ROS formation was assessed using fluorescence by means of the DCFH-DA method (information on relative amounts of oxygen radicals without selectivity), the Ghormley’s triiodide method using UV–vis spectrophotometry (concentrations of H_2_O_2_), and electron paramagnetic resonance with DMPO as the spin-trap agent (information on specific oxygen radicals). This approach elucidates that Cu NPs undergo ROS-generating corrosion reactions, which previously have not been assessed *in situ*. In the presence of H_2_O_2_, and based on the type of oxygen radical formed, it was concluded that released copper participates in Haber–Weiss and/or Fenton reactions rather than in Fenton-like reactions. The new combination of techniques used to determine ROS induced by Me NPs provides a way forward to gain a mechanistic understanding of Me NP-induced ROS formation, which is important for gaining crucial insight into their ability to induce oxidative stress.

## Introduction

Metallic nanoparticles (Me NPs) often behave differently than their corresponding micrometer-sized particles and massive surfaces. This is predominantly due to an increased surface-to-mass ratio and quantum confinement. From the rapid increase in the use of Me NPs in new applications and consumer products on the market and nonintentionally formed NPs at different occupational settings, e.g., during manufacturing, postprocessing, welding, combustion, etc., follows an obvious increased risk of their diffuse emissions and exposure to humans (and the environment).

Such exposure, mainly via inhalation and skin contact, may depend on characteristics, dose, and exposure setting, resulting in negative consequences for humans, such as inflammation and lung diseases. This requires the ability to predict the toxic potency of such Me NP exposure and that the underlying mechanisms be revealed and possibly linked to the physicochemical particle properties. The role of reactive oxygen species, ROS, formation on oxidative stress induced by exposure to NPs, including Me NPs, has been extensively studied, e.g., using protein signaling methods.^1^ Less studied is the importance of surface reactions that occur on NPs and in solution in biologically relevant media.

ROS (reactive oxygen species) is a collective term for oxygen species with higher reactivity than O_2_, such as H_2_O_2_, HO^•^, O_2_^•–^, HOO^•^, and O_2_^1^. These species can be formed by organelles both naturally and in response to Me NP exposure. Superoxide (O_2_^•–^) and hydrogen peroxide (H_2_O_2_) are examples of reactive species produced naturally during metabolic reactions in both mitochondria and peroxisomes (organelles).^2,3^ The concentration of ROS varies in the body, with endogenous concentrations of H_2_O_2_ in blood between 0.8 and 6 μM,^4^ <0.9 μM in breath condensate, and tens of μM in the alveolar lining fluid.^5^ In excess of ROS, e.g., as a result of Me NP exposure, cells may experience oxidative stress.^6−9^ Me NP-induced ROS-generating reactions at the particle surface and in solution, including Fenton, Fenton-like, Haber–Weiss, electrochemical, photoinduced, and defect-induced reactions, were recently summarized by the authors in a review paper, and the importance of chemical speciation of released metals and of redox characteristics of metal oxides was emphasized.^10^ Electrochemical corrosion reactions of metal NPs (core–shell NPs) are highly metal-specific and can result in ROS formation. In addition, Me NP–biomolecule interactions will affect the ROS-producing mechanisms and reactions that take place at the NP surface and/or as secondary reactions in solution.

A multianalytical approach, combining multiple techniques and methods, is essential for comprehensively understanding the mechanisms of Me NP-induced ROS formation. It allows researchers to investigate these complex processes from various angles and build a more holistic understanding of the potential risks and impacts associated with Me NPs.

Different assays, such as the DCFH-DA (2′,7′-dichlorofluorescein diacetate) assay with and without HRP (horseradish peroxidase), are available to assess ROS levels in biological settings.^11^ This method has been widely used in nanotoxicology to detect acellular ROS production from NPs since it produces clearly visible fluorescence images and is easy to perform and both efficient and cost-effective. The DCFH assay is activated by the presence of the HO^•^, HOO^•^, and O_2_^•–^ oxygen radicals but not by H_2_O_2_, which forms a fluorescent DFC^•^ molecule and is measured using fluorescence spectroscopy. Detection of H_2_O_2_ requires the addition of HRP (the horseradish peroxidase enzyme). The analyses have some drawbacks due to the self-initiation of DCFH but are still commonly used due to the simplicity using the assay. The addition of HRP in solutions with Me NPs has recently been shown to give incorrect readings due to HRP-Me NP interactions.^12^ Another disadvantage of the DCFH assay is its photosensitivity^13^ and lack of specificity on which specific radicals that activate DCFH.

**Figure 1:**
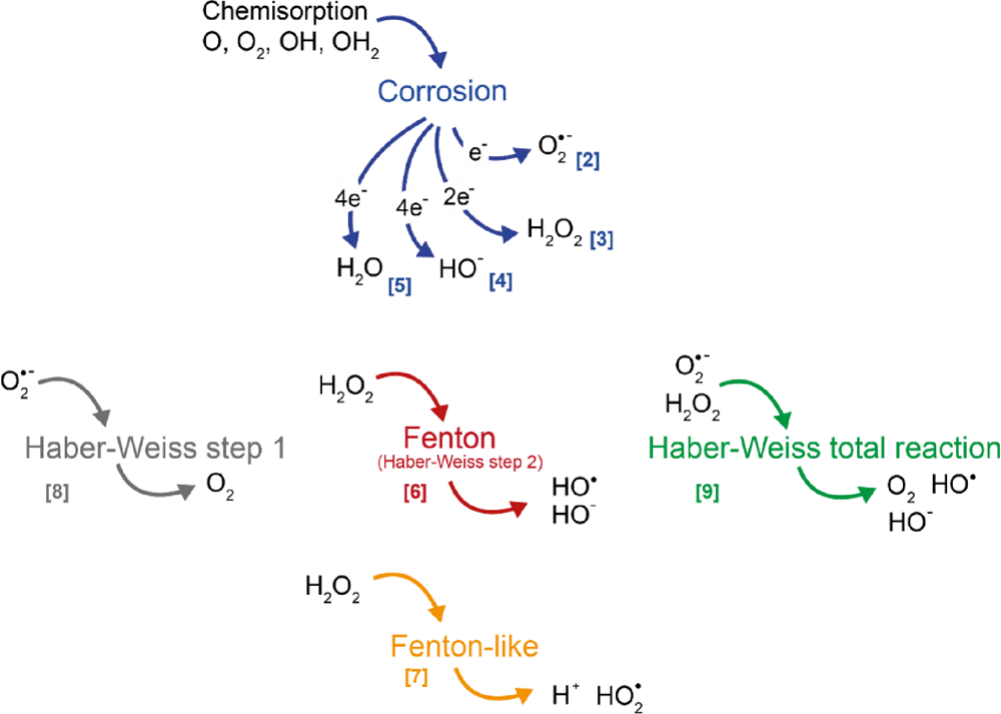
ROS mechanisms of interest to metal NPs. Reduction reactions appear as Fenton, Fenton-like, and corrosion reactions, all accompanied by metal oxidation. The first stage of the Haber–Weiss reaction is an oxidation reaction that reduces the concentration of a metal. The total Haber–Weiss reaction includes the Fenton reaction, which reoxidizes the metal, resulting in no net change in the oxidation state of the metal. The numbers in square brackets refer to the different reactions given below.

The objective of this study was to explore the possibility of using complementary techniques to the DFCH-DA assay for ROS measurements to enable *in situ* detection of specific oxygen reactive species and of H_2_O_2_, combined with biodissolution measurements, using electron paramagnetic resonance (EPR) (to obtain specific information about oxygen radicals) and the Ghormley’s triiodide method (to specifically detect and measure the concentration of H_2_O_2_) using UV–vis. The multianalytical approach was employed on Cu NPs in PBS. Cu NPs were investigated because they have been extensively studied from a biodissolution/transformation and ROS formation perspective and because of the fact that they are diamagnetic, removing the risk of the particles interfering with the EPR reading.^14−19^ PBS was chosen because of its simplicity and pH (7.4), which is similar to physiological conditions.^20^ The reaction mechanisms of interest to the investigated system are listed in Figure 1.

According to literature findings, Cu NPs can undergo corrosion, Fenton, Fenton-like, and Haber–Weiss reactions.^21^ Their respective designation used in this study is illustrated in Figure 1 and reactions [1–9]. Since the denomination of prevailing reaction mechanism is used differently in the literature, e.g., the total Haber–Weiss reaction is written as the combination of the metal oxidating Fenton [6] and the metal reducing Haber–Weiss reaction [8]. In the following, the Haber–Weiss reaction refers to the total reaction [9].
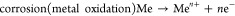
1
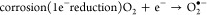
2
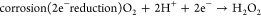
3
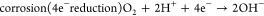
4
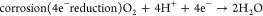
5
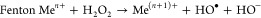
6
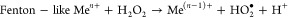
7
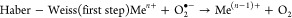
8
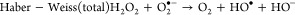
9

The authors have previously suggested that metal and metal oxide NPs can be grouped into different tiers, Table 1, depending on their respective ROS generating mechanisms.^10^ If Cu NPs can undergo Fenton or Fenton-like, Haber–Weiss, and any of the corrosion reactions, as mentioned in the previous literature, it would place Cu NPs in the first tier.

**Table 1:** Proposed Categorization of Me NPs Based on ROS-Formation Mechanisms (Adapted from Reference (10))

tier 1	tier 2	tier 3	tier 4
surface catalysis	surface catalysis	surface catalysis	surface catalysis
band gap redox	band gap redox	band gap redox	
Haber–Weiss	Haber–Weiss		
Fenton	Fenton		
Fenton(-like)	Fenton(-like)		
corrosion			

Since the HRP enzyme has been shown to adsorb to the Cu NPs, and thus interfere with the DCFH-HRP measurements, the method should not be used to evaluate the presence/formation of H_2_O_2_.^12^ This was instead done by using Ghormley’s triiodide method, where the iodine ion reacts with H_2_O_2_ to form the triiodide ion, which is yellow [10]. Its concentration, based on calibration curves, can therefore be indirectly determined based on the rate of degradation using UV–vis (350 nm).
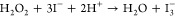
10

If H_2_O_2_ is added into, or formed, in a system containing Me NPs that, via different reactions, can undergo Fenton or Fenton-like reactions, the kinetics of H_2_O_2_ consumption can be determined.^22^ The disadvantage of Ghormley’s method is that it cannot provide information about other types of ROS present in the system.

ROS species are predominantly radicals, making electron paramagnetic resonance (EPR) suitable for their detection. However, due to their extremely short lifespan, they are difficult to detect. To circumvent this obstacle, spin-traps have been widely applied in the field. DMPO (5,5-dimethyl-1-pyrroline-*N*-oxide), a cyclic nitroso compound commonly used in trapping ROS, reacts easily with a ROS radical, forming a spin-adduct in the form of a DMPO-ROS radical to be measurable in EPR. The fact that spin-adducts are stable radicals and that their characteristic EPR spectra report the origin of ROS specifically makes this technique very powerful. EPR measurements were therefore carried out to provide information on specific radical formation as this information would allow predictions of the underlying ROS reaction mechanisms of a given Me NPs, thus allowing its categorization into the different tiers suggested above as a first screening step to assess the potency of Me NP-induced oxidative stress.^10^ In future studies, other spin-traps such as BMPO (5-*tert*-butoxycarbonyl-5-methyl-1-pyrroline-*N*-oxide) and DEPMPO (5,5-dimethyl-1-pyrroline *N*-oxide) should be evaluated and selected based on the stability and half-life of the spin-adducts of interest.^23^ A disadvantage with the EPR method is that paramagnetic metals (the majority of the transition metals) and species can interfere with the readings, and the metal signal can overlap with the signal of the specific spin-adduct of interest.

## Experimental Section

### Materials and Exposure Solutions

Cu metal NPs were purchased from American Elements, Los Angeles, CA, USA (average particle size <100 nm, purity >99.9%). The NPs were cleaned in ethanol for 5 min using an ultrasonic probe (Branson Sonifier 250), followed by drying at 80 °C for 2 h before being stored for a minimum of 24 h in a desiccator prior to use. Cleaning was carried out to remove any organic impurities that could interfere with the analysis. The short sonication time was selected to limit the effect of sonication on particle dissolution.^24^ A Cu NP stock solution was prepared of 1 mg cleaned Cu NPs/mL PBS (phosphate buffered saline) followed by vortexing for 10 s, sonication for 10 min in an ultrasonic bath (230 V/50–60 Hz180VA, VWR ultrasonic cleaner, Malaysia), and vortexing for 10 s. This cycle was repeated twice.

PBS was prepared using 8.77 g/L NaCl (VWR Chemicals, AnalaR Normapur), 1.28 g/L Na_2_HPO_4_, 1.36 g/L KH_2_PO_4_ (EMSURE, anhydrous for analysis), 370 μL/L 50% NaOH (Emsure, 50%, for analysis), and ultrapure water (18.2 MΩ cm, MilliPore, Solna, Sweden. The pH was adjusted to 7.4 (PHM210, MeterLab, Radiometer analytical). H_2_O_2_ (Suprapur, 30%, Merck Darmstadt, Germany) was diluted in PBS immediately before use in dark vials. All analyses and sample preparations were carried out in dark conditions at room temperature.

**Figure 2:**
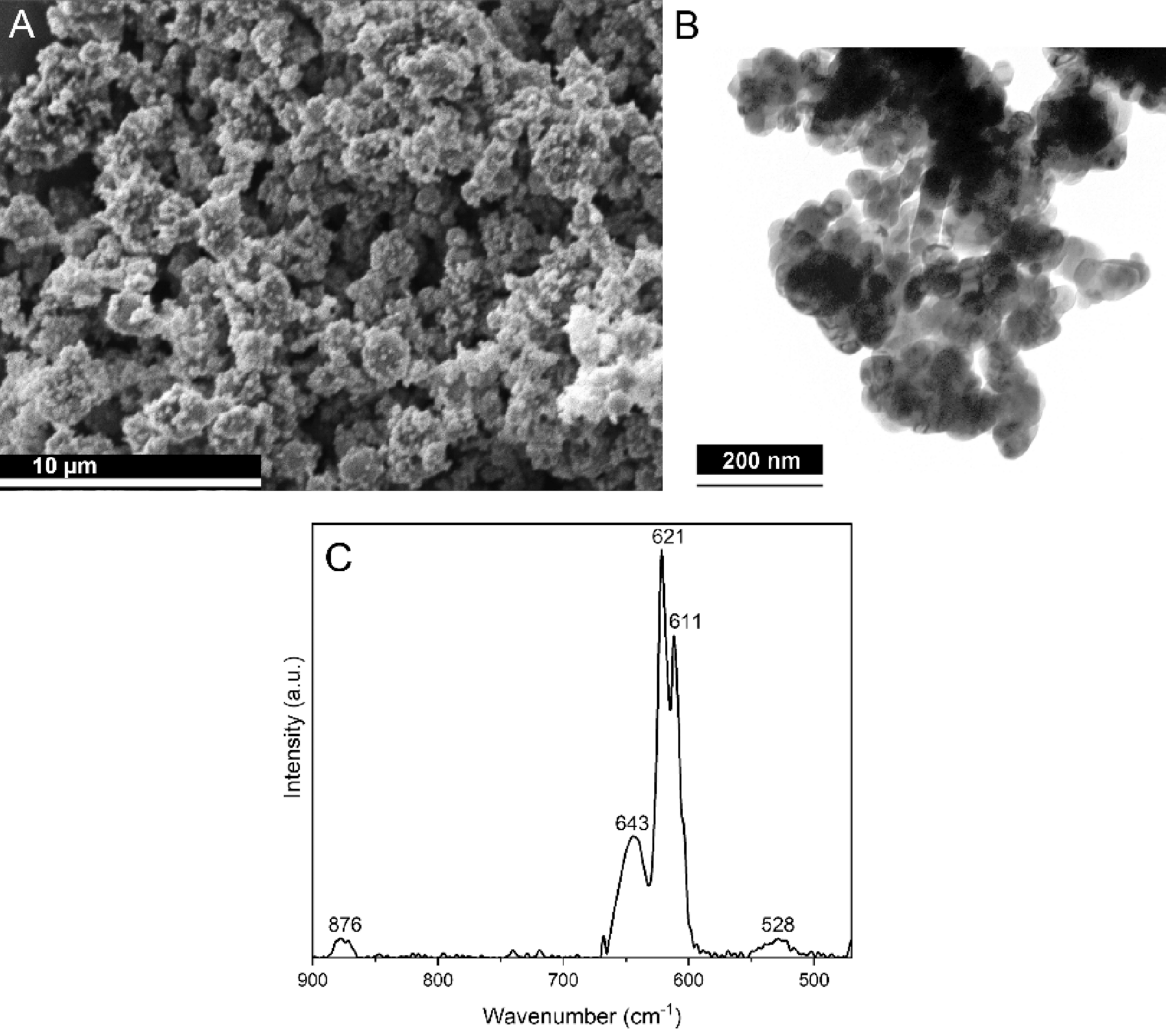
Cu NP characteristics, morphology, and size distribution visualized with SEM (A) and TEM (B), and surface composition determined using FTIR (C).

Particle characteristics in terms of particle size distribution and primary sizes were determined using scanning electron microscopy and SEM (XL30 ESEM, ThermoFisher Scientific, 20 kV) and transmission electron microscopy, TEM (HT7700 Hitachi instrument, 100 kV). In short, particle/aggregate sizes ranged between 2 and 220 nm (SEM), with primary particle sizes ranging between 2 and 70 nm (TEM), Figure 2A,B. Dry powder of NPs was applied to carbon tape for the SEM investigation. TEM samples were prepared by sonication of the NPs in ethanol for 15 min before being applied to a 200-mesh copper grid with Formvar/carbon support films (Ted Pella, Inc.), followed by storage in a desiccator for 24 h before analysis.

Fourier transform infrared spectroscopy, FTIR, using a Bruker Tensor 37 instrument equipped with a DTGS detector, was performed on pressed CuNP/KBr tablets (0.6 mg of Cu NPs/200 mg of KBr) to determine their surface oxide composition. The results are presented in Figure 2C. The high-intensity bands between 645 and 610 cm^–1^ correspond to the Cu(I)–O bond, indicating the predominance of Cu_2_O.^25−29^ Small amounts of CuO could be distinguished following the interpretation from the low intensity band around 530 cm^–1^, which corresponds to Cu(II)–O.^25^ The low-intensity band around 877 cm^–1^ was assigned to Cu–O–H.^29^

The extent of copper release from the NPs exposed in PBS for 1 h with and without H_2_O_2_ (0, 2.5, and 100 μM) was determined. The exposed particles were separated using an ultracentrifuge (Beckman Optima L-90K ultracentrifuge, 50,000 rpm, 1 h). The supernatant was digested with 0.49 M H_2_O_2_, 0.07 M HNO_3_, and MQ-water for 35 min using UV light (705 UV Digester, Metrohm) before being analyzed by means of atomic absorption spectroscopy, AAS (PerkinElmer AA800 analyst instrument). Dose samples were analyzed by complete Cu NP dissolution using concentrated aqua regia (3:1, 38% HCl: 68% HNO_3_) for 24 h, showing that the administered particle dose was 44 μg/mL, compared to the nominal particle dose (100 μg/L). Lower administered doses compared to the nominal doses of Me NPs are consistent with previous investigations.^24^ The detection and quantification limits were 10 μg Cu/L.

The extent of particle dissolution, Table 2 and Figure S1, was found to be somewhat inhibited in the presence of low amounts of H_2_O_2_ (2.5 μM), while the released fraction was the same for the high H_2_O_2_ concentration (100 μM) observed without H_2_O_2_. This is in line with literature findings showing that H_2_O_2_ promotes copper oxide formation, which improves the barrier properties.^30^ The reason this was not observed for the highest H_2_O_2_ concentration remains to be explored.

**Table 2:** Summary of the Characterization Analysis of the Cu NPs

information	analytical technique	results		
copper release (1 h)	AAS	no H_2_O_2_	2.5 μM H_2_O_2_	100 μM H_2_O_2_
		5.6 ± 0.1%	2.2 ± 0.6%	5.5 ± 0.1%
surface oxides	FTIR	Cu_2_O, CuO, CuOH		
primary NP size	TEM	2–70 nm		
agglomerate size	SEM	7–220 nm		

### Methods for ROS Detection

#### 2′,7′-Dichlorofluorescein Diacetate, DCFH-DA, Assay

The fluorescence of DCF• was determined using an Infinite F200 PRO multimode plate reader (Tecan, Austria). The excitation wavelength was set to 485 nm, and the emission wavelength to 535 nm. Each well was filled with a total volume of 200 μL containing either 0 or 100 μg/mL Cu NPs, with 0, 0.0025, 0.005, or 5 mM H_2_O_2_, and 0 or 2.2 u/mL HRP (Sigma-Aldrich, type II, essentially salt-free, lyophilized powder), 0.015 mM DCFH-DA (Sigma-Aldrich, ≥ 97%), and 0.54 mM DMSO (dimethyl sulfoxide, Sigma-Aldrich, ReagentPlus, ≥99.5%). Prior to mixing, the DCFH-DA was dissolved in DMSO and deacetylated (i.e., removing DA) with 0.01 M NaOH for 30 min in dark conditions.

The results were averaged and divided by the signal ratio between the sample and blank intensity. The 2.2 u/mL HRP corresponds to 2.2 μmol/min of catalytic conversion. Thus, saturation should be reached at 2.5 μM H_2_O_2_, which was the lowest concentration chosen. Since HRP ideally regenerates, the second concentration was twice as high, i.e., 5 μM H_2_O_2_. A significantly higher concentration of H_2_O_2_, 100 μM, was also investigated to determine the effects of a supersaturated system.

#### Ghormley Triiodide Method

H_2_O_2_ concentrations were determined by measuring the absorbance at 350 nm using a UV–vis spectrophotometer (JENWAY 6300, Crelab, Sweden) according to the Ghormley triiodide method. All experiments were conducted under dark conditions to prevent any photoactivation of the system. The absorbance was converted to concentration using calibration curves (0, 2.5, 5, 50, 100, and 500 μM H_2_O_2_ in PBS) produced directly before the measurements. Each sample was prepared immediately before analysis in a quartz cuvette. Solutions of 0.1 mL of potassium iodide (KI) and solutions with 0.5 M sodium acetate (NaAc), 0.5 M acetic acid (Hac) (1 M NaAc/Hac buffer), and 1.6 mM ammonium dimolybdate (ADM), acting as a catalyst for the oxidation of iodine, were prepared in parallel. The final samples contained 0.05 M KI, 0.05 M NaAc/Hac, and 0.8 mM ADM with 0, 20, or 100 μg/mL Cu NPs, and 0, 2.5, 5, 50, or 100 μM H_2_O_2_. The selected H_2_O_2_ concentrations were the same lower values as those used in the DCFH method and the higher concentrations of the calibration curve. Calibration curves are available in the Supporting Information (Figure S2).

#### Electron Paramagnetic Resonance, EPR

Electron paramagnetic resonance (EPR) spectroscopy analysis was performed on a Bruker EMX-micro spectrometer equipped with an EMX-Primium bridge and an ER4119HS resonator and controlled with Bruker Xenon software. DMPO (5,5-dimethyl-1-pyrroline-N-oxide, ≥ 97%, Sigma-Aldrich) was used as a spin-trap for ROS radicals. As previously described, a stock solution of Cu NPs was prepared and mixed with DMPO and H_2_O_2_. The final concentrations were 100 μg/mL Cu NPs, 0.1 mM DMPO, and 0, 0.0025, 0.1, or 5 mM H_2_O_2_. The solutions were vortexed and allowed to react for approximately 60 s before being transferred to a glass capillary (Hirschmann, Germany, Na-heparin capillary for blood gas analysis, diameter: 1.75 mm, 75 mm/100 μL). Thin capillaries are necessary for the analysis of an aqueous solution due to the high dielectric constant of the H_2_O molecules. When loading a sample, the capillary was placed at the top of an Eppendorf tube to limit the number of dispersed particles entering the capillary. It was then inserted into the test chamber of the resonator. The capillaries were open at both ends to assist the capillary forces but sealed using polymer clay after sample loading.

In this experiment, the sedimentation rate of the Cu NPs was sufficient to minimize particles entering the capillary. In future studies with paramagnetic NPs with slow sedimentation rates, it could be necessary to centrifuge or filter the sample before loading it into the capillary. The first spectrum was acquired 2 min after sample mixing, followed by two more recordings at 5 min intervals. The main settings were adjusted with PBS only as presets before the sample preparation to minimize the time required for fine-tuning after sample insertion into the resonator. The final recording conditions were set as follows: microwave frequency of 9.86 GHz at a power of 5 mW, and a modulation frequency of 100 kHz with an amplitude of 0.3 mT. These conditions were employed for all spectra as well as for data fitting (see fitting example in Figure S3). The spectral resolution was gained at a time constant of 10.24 ms for 1024 data points. To retrieve the spectra parameters, the EasySpin software packet (version easyspin-6.0.0-dev.34)^31,32^ was used. More specifically, the esfit-function “garlic” was used for all spectral simulations and fitting in this work. Fitting parameters for each species included the *g*-value in combination with hyperfine coupling constants arising from the nuclear spin of nitrogen atom and superhyperfine coupling constant(s) arising from proton(s).

## Results and Discussion

### ROS Measurements Using the DCFH Assay with and without HRP

**Figure 3:**
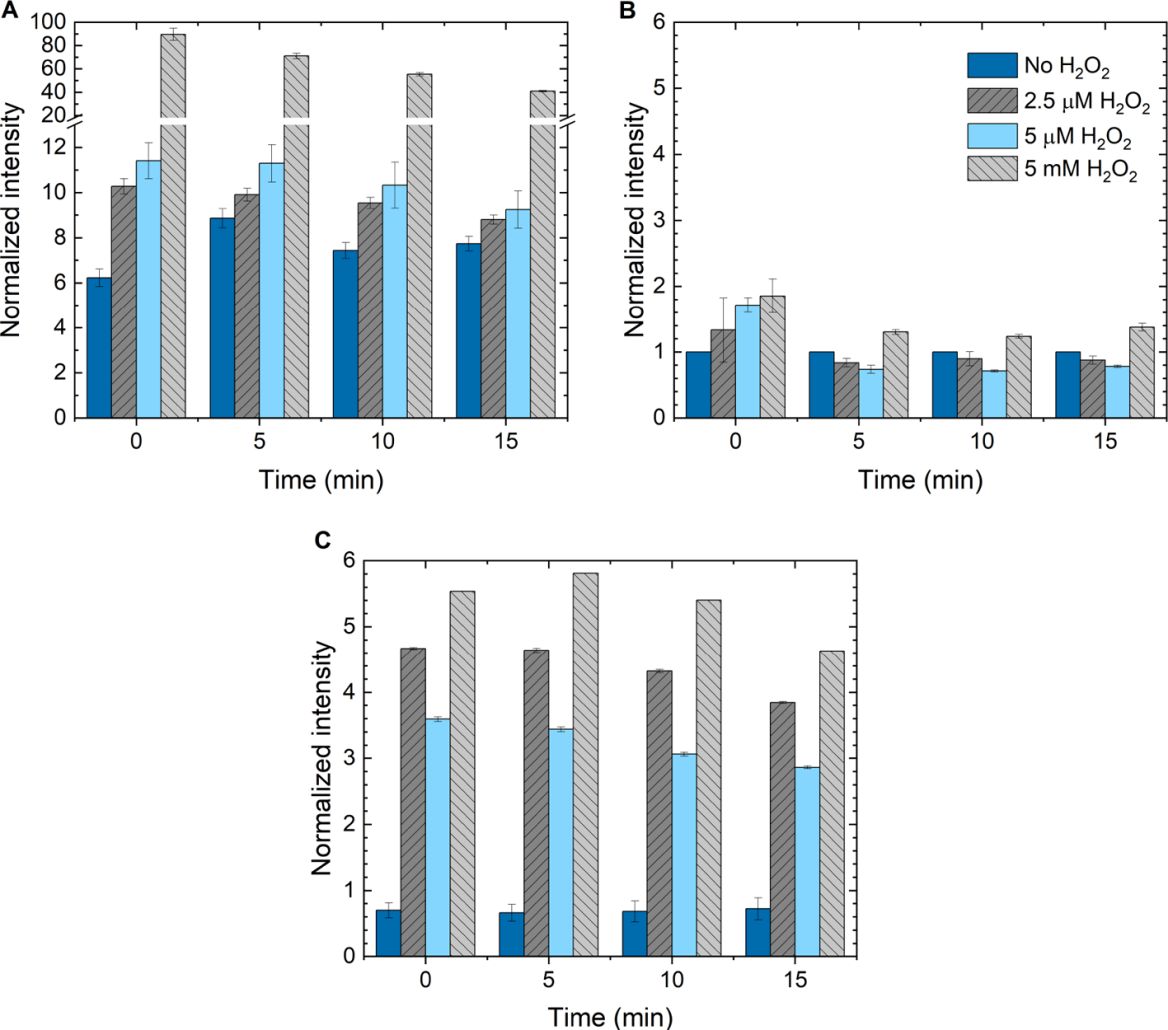
ROS measurements of Cu NPs in PBS using the DCFH method with and without HRP and the addition of H_2_O_2_ at different concentrations. The results are presented as the intensity for the samples with Cu NPs normalized to the signal of PBS only (no Cu NPs) after each exposure time. (A) Cu NPs were prepared in PBS without HRP for various added H_2_O_2_ concentrations. (B) PBS without Cu NPs or HRP for different added H_2_O_2_ concentrations. (C) Cu NPs in PBS with HRP for different added H_2_O_2_ concentrations.

The results of the ROS measurements using the fluorescent DCFH assay with and without HRP are presented in Figure 3. The presence of Cu NPs in PBS without any H_2_O_2_ addition (Figure 3A) resulted in ROS formation, i.e., 6–8 increased levels, compared to the blank solutions (Figure 3B). Immediately after mixing, the signal from the samples with added H_2_O_2_ surpassed those without H_2_O_2_. Upon addition of H_2_O_2_ (Figure 3A) the ROS levels increased approximately 2-fold for the lowest H_2_O_2_ concentration (2.5 μM H_2_O_2_), 2.5-fold higher for the 5 μM H_2_O_2_ concentration, whereas the 1 order of magnitude higher H_2_O_2_ concentration, 5 mM, was almost 90 times higher.

Increased ROS formation in the presence of Cu NPs compared to samples without NPs show the formation of ROS, either HO^•^, HOO^•^, and/or O_2_^•–^ radicals as they activate DCFH by hydrogen abstraction. This implies Fenton-, Fenton-like, Haber–Weiss, and/or 1-electron transfer corrosion reactions taking place in solution with released copper from the Cu NPs, though any differentiation between the mechanisms cannot be made. Samples with the highest concentration of added H_2_O_2_ showed a reduced ROS response over time. This reduction over time was attributed to the consumption of both DCFH and H_2_O_2_. Similar, albeit nonstatistically significant, trends were observed for the Cu NP samples with no or low concentrations of added H_2_O_2_. The Cu NP samples with low concentrations of added H_2_O_2_ showed after 15 min no significant difference with the samples without H_2_O_2_. This elucidates that H_2_O_2_ is not reactive enough to active DCFH. However, the natural decay of H_2_O_2_ with time, forming HO^•^ could possibly activate DCFH.

HRP was added to the Cu NP-PBS solutions as the enzyme since it enabled ROS measurements of H_2_O_2_. The results are presented in Figure 3C. Previous investigations have shown that HRP can adsorb onto the Cu NPs and thus become unreactive.^12^ All solutions with Cu NPs without any H_2_O_2_ addition showed lower signals compared to the PBS and HRP solution only (<1 in normalized intensity), Figure 3C. This supports the conclusion that the presence of Cu NPs in combination with HRP results in HRP deactivation and, thus, incorrect ROS measurements. The ROS signal does not appear to have a linear correlation with added H_2_O_2_ concentration, which is speculated to be due to changes in the release and passivation behavior.^30^ The results support the earlier claim that HRP addition to the DCFH assay should be used with caution when assessing ROS production by Me NPs, since it, to varying extents, can adsorb to the Me NPs and hamper the results.^12^

### Formation and Degradation of H_2_O_2_ Investigated by Ghormely’s Triiodide Method

**Figure 4:**
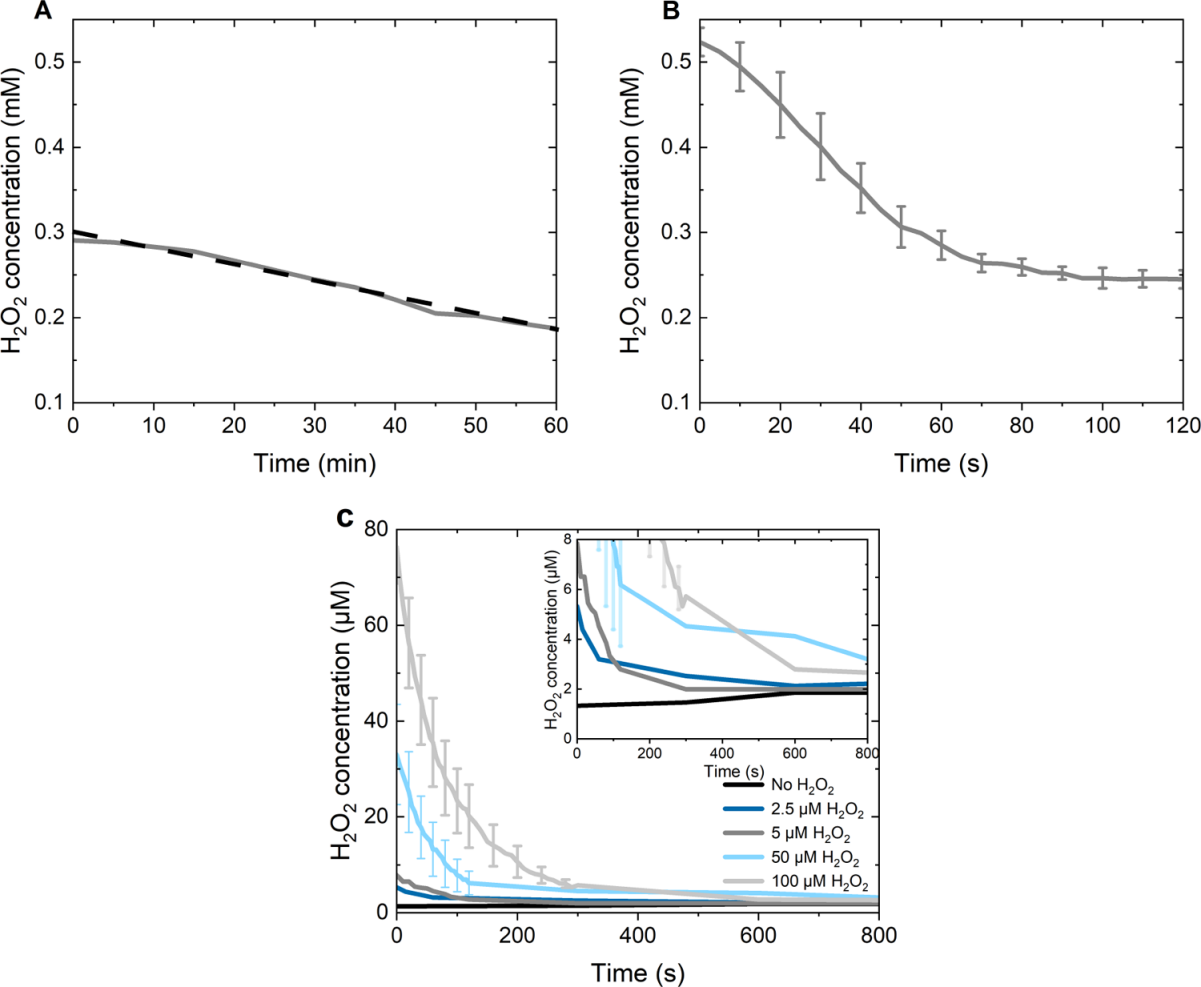
Measurements of H_2_O_2_ degradation and formation in the presence of Cu NPs in PBS with and without the addition of H_2_O_2_ using Ghormley’s triiodide method. (A) Cu NPs (100 μg/mL) with no added H_2_O_2,_ dashed line shows a linear fitting (*R*^2^ = 0.98); (B) Cu NPs (100 μg/mL) and 1 mM H_2_O_2_; (C) Cu NPs (20 μg/mL) in PBS with and without the addition of H_2_O_2_ (0, 2.5, 5, 50, and 100 μM). The standard deviations reflect two unique measurements.

Since the DCFH method with HRP can impede measurements of ROS formation, including H_2_O_2_ in the presence of Me NPs, the applicability of Ghormley’s triiodide method^33^ to determine H_2_O_2_ concentrations in solution were investigated. Investigations were conducted for two different concentrations of Cu NPs (20 and 100 μg/mL) in PBS with and without the addition of H_2_O_2_ (0, 2.5, 5, 50, and 100 μM). The results are presented in Figure 4, showing both H_2_O_2_ degradation and formation.

The measurements for both Cu NP concentrations in PBS (without added H_2_O_2_) indicated the formation of H_2_O_2_ formation. The higher particle loading revealed the presence of H_2_O_2_ in the system already upon data collection, Figure 4C, while the kinetics of H_2_O_2_ formation for the lower particle concentration was slower and possible to observe with an increase of the H_2_O_2_ concentration by ∼40% within the time period examined (5 min), inset in Figure 4C. Consumption of H_2_O_2_ was evident for both particle concentrations. The H_2_O_2_ equivalent decreased over time for both particle concentrations.

A difference could be seen between the added (theoretical) concentration and the measured H_2_O_2_ equivalent at the first data point. This is explained by reactions that take place prior to commencing data collection. These reactions can both be degradation reactions such as Fenton, Fenton-like, or Haber–Weiss reactions, seen for the samples with higher added H_2_O_2_ concentration (>50 μM), and formation reactions such as via the 2-electron transfer corrosion reaction, seen for samples with lower (<5 μM) or no added H_2_O_2_ (Table 3, highlighted in light gray). The rapid degradation of H_2_O_2_, seen in Figure 4 and Table 3, could possibly explain why no passivation effects, from a copper release perspective, were observed in the presence of high concentrations (100 μM) of H_2_O_2_, see Table 1.

**Table 3:** Summary of Changes in H_2_O_2_ Equivalents from Figure 4^a^

Cu NPs (μg/mL)	added **H**_**2**_**O**_**2**_ (μM)	first data point (μM)	change first to last data point (%)	change first to last data point (μM)	time (min)
100	0	291	–36	–104	60
100	1000	523	–53	–278	2
20	0	1.9	+26	+0.5	10
20	2.5	5.3	–60	–3.2	10
20	5	7.8	–76	–5.9	10
20	50	33.0	–90	–28.9	10
20	100	76.3	–96	–73.5	10

a The added (theoretical) concentration of H_2_O_2_ differed from that of the measured H_2_O_2_ equivalent at the first data point. This is due to rapid reactions taking place prior to the first data collection; these could be both formation and degradation of H_2_O_2_.

Control experiments were performed to rule out the possibility that the Cu NPs, despite experiments under dark conditions, would absorb light and incorrectly indicate the presence of H_2_O_2_. This was confirmed since the measured absorbance for the Cu NPs in PBS without any addition of KI was less than one tenth of the lowest measured intensity from the Cu NPs in the KI solution. The absence of an eventual instrumental drift was assured by measuring standards before and after the measurements (see Figure S2). These control measurements confirmed that the observed increase in absorbance reflects the formation of H_2_O_2_. This implies that the Cu NPs corrode in the system, forming H_2_O_2_ in solution, as illustrated in Figure 1.

**Figure 5:**
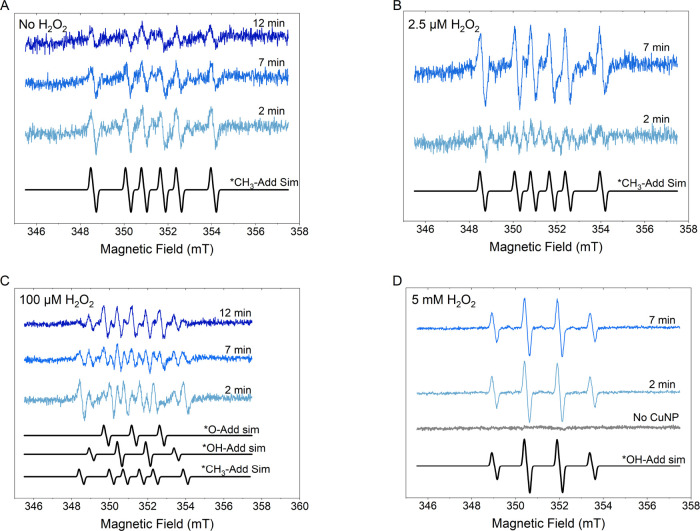
Time-dependent EPR results of Cu NPs in PBS with and without the addition of H_2_O_2_ addition. The blue lines are experimental results, and the lower black lines simulated spectra of different adducts, respectively: DMPO-HO^•^, DMPO-O_2_^•–^, and DMPO–CH_3_^•^. (A) No H_2_O_2_, (B): 2.5 μM H_2_O_2_, (C) 100 μM H_2_O_2_, and (D) 5 mM H_2_O_2_.

The degradation of H_2_O_2_ in solution was also investigated for the Cu NP-PBS system by adding known amounts of H_2_O_2_, Figure 4C. The first collected data points are equal to a lower H_2_O_2_ equivalent than the known quantity added to each sample. This difference is attributed to a rapid consumption of H_2_O_2_ taking place before the first data recording (∼10 s). All samples with H_2_O_2_ addition showed reduced absorbance over time, indicating H_2_O_2_ degradation via Fenton, Fenton-like, or Haber Weiss reactions, Figure 1 and [5], [6], and [8].

### Determination of Radical Species Formation Using the EPR Technique

Since the DCFH assay provides information on ROS but cannot distinguish between the various reactive oxygen radicals (HO^•^, HOO^•^, and O_2_^•–^) and Ghormely’s method only provides information about H_2_O_2_ formation/degradation without any information on reaction products, the EPR technique was implemented. This technique provides information about the formation of radical species and, thus, about prevailing ROS formation mechanisms. This knowledge can help to categorize Me NPs (Table 1) as a first step in predicting the toxic potency of Me NPs to induce oxidative stress.^10,34^

The results of the EPR measurements are shown in Figure 5A–D. Generally, the recorded EPR spectra showed a rich hyperfine splitting pattern. Identification of each of the obtained spin-adducts was successfully carried out by recognizing the adapted parameters in good accordance with the corresponding literature values. Thus, three types of DMPO-radical spin-adducts were found among all Cu NP-PBS samples at varied H_2_O_2_ concentration ranging between 0 and 5000 μM, and their simulated spectra are exampled at the lower part of Figure 5C. In the presence of a high concentration of H_2_O_2_, e.g., 5,000 μM, the formation of DMPO-HO^•^ adduct (*g* = 2.0059, *a*_N_ = 41.929 MHz, *a*_H(β)_ = 41.435 MHz) dominated, and the product appeared stable over the measuring time, Figure 5D.

At lower H_2_O_2_ concentrations, however, e.g., 2.5 and 100 μM, the DMPO–CH_3_^•^ (*g* = 2.0058, *a*_N_ = 41.654 MHz, *a*_H(β)_ = 69.780 MHz) was detected at a varied ratio, Figure 5B,C, respectively. In particular, this species was found even without H_2_O_2_, Figure 5A. Since the samples did not contain any organic methylate component, its presence is interpreted as a result of DMPO degradation, most probably catalyzed by the Cu NPs. That degradation of DMPO in the presence of Cu leads to a release of CH_3_^•^ has been reported earlier in the literature.^35^ The rate of this reaction seemed to be accelerated in the presence of low H_2_O_2_ concentration, e.g., 2.5 μM (Figure 5B), where the production of DMPO-HO^•^ probably is very low. The DMPO–CH_3_^•^ spin-adduct appeared semistable, as it disintegrates within minutes of the time scale, clearly seen in Figure 5A. This decaying behavior was also reported in the previous study.^35^ Moreover, CH_3_^•^ production did not occur in the absence of Cu NPs (Figure 5D). It was first observed when Cu NPs were present in the system. It follows that the tentative degradation of DMPO must be a consequence of the Cu NP’s reactivity. The fact that DMPO–CH_3_^•^, or, for that matter, any other spin-adducts except DMPO-HO^•^, did not appear in Figure 5D despite the presence of Cu NPs could be rationalized by a rapid formation of DMPO-HO^•^ at high concentrations of H_2_O_2_, accompanied by its good stability. Hence, its intense EPR signal might cover all other, if any, radicals formed.

Interestingly, at moderate H_2_O_2_ concentration, e.g., 100 μM, three types of DMPO radical adducts were detected simultaneously at the first EPR read, which was ∼2 min after the Cu NP-PBS mixture solution was prepared. All were identified by spectral fitting and degradation (see the bottom part of Figure 5C). In addition to the aforementioned stable DMPO-HO^•^ and decaying DMPO–CH_3_^•^, a third component was found to be a DMPO-O_2_^•–^ spin-adduct (*g* = 2.0059, *a*_N_ = 41.408 MHz, *a*_H(β)_ = 13.304 MHz). Corrosion of Cu NPs can result in the following reaction: (1) O_2_^•–^ [1]. Apart from the stable DMPO-HO^•^ and the decaying DMPO–CH_3_^•^ species, the DMPO-O_2_^•–^ spin-adduct evolved to become more intense over time from 7 to 12 min, Figure 5C, indicating radical formation. Slow oxygen diffusion into the capillary over time cannot be excluded.

In conclusion, although Cu corrosion and DMPO degradation due to the reactivity of Cu NPs were confirmed, the formations of DMPO-O_2_^•–^ and DMPO–CH_3_^•^ are considered to be formed to a minor extent in the presence of H_2_O_2_. The main reaction of Cu NPs with H_2_O_2_ is the rapid formation of the ROS species, HO^•^, displayed via its spin-adduct DMPO-HO^•^, whose EPR signal was easily detectable and stable. This conclusion is supported by the UV–vis measurements presented in Figure 3. Therefore, it is inferred that either Fenton or possibly Haber–Weiss reactions (see Figure 1) and [6] and [9] participate in this system.

## Summary – Cu NP-Induced ROS Formation Mechanisms

Information from complementary techniques, including the DFCH-DA assay for ROS measurements of hydroxyl, peroxyl, and other oxygen radicals, Ghormely’s method to assess H_2_O_2_ formation/degradation, and EPR to assess specific radicals in solution, was combined in this study to provide an improved understanding of acellular ROS formation mechanisms induced by Me NPs. This was elucidated for Cu NPs in PBS with and without H_2_O_2_.

Literature findings show that Cu surfaces are known to break down H_2_O_2_ in aqueous solutions, primarily via Fenton or Fenton-like reactions.^36^ Fenton or Fenton-like reactions are sometimes described as being interchangeable. This is incorrect because their respective reaction results in different reaction products.^10^ The prevailing ROS reaction can be assessed by analyzing these products; HO^•^ and HO^–^ are formed via the Fenton reaction, while H^+^ and HOO^•^ are formed in a Fenton-like reaction.^37^ HO^•^ and HO^–^ can also be formed via Haber Weiss reactions.^37^ However, few techniques are able to determine these species due to the high reaction rates and often low concentrations.

ROS measurements using the DCFH assay clearly revealed the formation of reactive oxygen species for the Cu NP-PBS system both with and without H_2_O_2_. The method cannot specify the specific radicals formed and hence not distinguish which ROS formation mechanisms prevail, which could be Fenton, Fenton-like, or Haber Weiss reactions. This type of measurement cannot provide information whether ROS is induced by corrosion reactions taking place on the surface of the Cu NPs.

Measurements using the Ghormley’s triiodide method showed that degradation of H_2_O_2_ took place in solution in the presence of the Cu NPs. This degradation can be a result of Fenton, Fenton-like, or Haber Weiss reactions. The method also showed that the Cu NPs produce H_2_O_2_ in PBS. The H_2_O_2_ equivalent concentration was shown to increase up to 10 min after sample preparation for Cu NPs (20 μg/mL) in PBS and be rapidly formed and present in the system already within 2 min, when the first spectrum was acquired, for the higher concentration of Cu NPs (100 μg/mL), followed by reduced concentrations (degradation) of H_2_O_2_ up to 60 min. Slower reaction rates of H_2_O_2_ formation were evident for the lower particle concentration (20 μg/mL), allowing kinetic information. Both measurements support the idea that Cu NPs corrode and form H_2_O_2_, i.e., via equation [3].

The results from Ghormley’s method investigation suggest that corrosion of the Cu NPs in PBS results in H_2_O_2_ formation. The EPR investigation concluded the presence of HO^•^, implies that H_2_O_2_ can be decomposed in solution via Fenton or Haber Weiss reactions. Although no DMPO-HOO^•^ EPR signal could be detected, the formation of DMPO-HOO^•^ and thus the possibility of a Fenton-like reaction mechanism cannot be ruled out by this study because the DMPO-HOO^•^ adduct is known to be short-lived, and it decays into DMPO-HO^•^. This transformation may well be accelerated by the reactivity of the Cu NPs and escape from EPR detection. Future studies should use other spin-traps, such as BMPO or DPEMPO, which are more specific to these radicals. DMPO can be oxidized by HO^•^ forming DMPO–CH_3_^•^ and DMPO-H adducts. The previous adduct was observed in most solutions (except the solution with 5000 μM H_2_O_2_) containing Cu NPs, probably due to DMPO oxidation or degradation, given that no other methyl-carrying organic compounds were present in the investigated solution.

Generated results using combined information on ROS formation using the DCFH assay (oxygen radicals), the Ghormley’s triiodide method (H_2_O_2_), and EPR (specific radical identification) show that Cu NPs in PBS corrode in solution via one and two electron reactions, [2] and [3], forming H_2_O_2_, which decomposes into mainly the HO^•^ free radical via Fenton and/or Haber Weiss reactions.

In summary, this study provides a better understanding of how Me NPs, here Cu NPs, result in ROS formation, known to induce oxidative stress, by using a combination of techniques able to provide specific information about the ROS species and the underlying reaction mechanisms. Such information is important for assessing health risks associated with exposure to Me NPs.

## Supporting Information Available

(Figure S1) Released amount of Cu per particle mass after 1 h of exposure in PBS with and without H_2_O_2_; (Figures S2 and S3) H_2_O_2_ calibration curves from measurements with Cu NPs (the Ghormley’s triiodide method); and (Figure S4) an example of EasySpin spectral fitting on the spectrum of Figure 5C (12 min), where three components of spin-adducts were identified (PDF)
