# Supplementary material for: Unravelling the Mechanistic Understanding of Metal Nanoparticle-Induced Reactive Oxygen Species Formation: Insights from a Cu Nanoparticle Study
Source: Chem Res Toxicol. 2023 Nov 10;36(12):1891–900. doi: 10.1021/acs.chemrestox.3c00177 (PMC10731636; doi:10.1021/acs.chemrestox.3c00177)
Supplement: Supplementary file 1 — tx3c00177_si_001.pdf [file tx3c00177_si_001.pdf]

Supporting Information

Unravelling the mechanistic understanding of metal nanoparticle-induced reactive oxygen species formation: Insights from a Cu nanoparticle study

Amanda Kessler[1], Ping Huang[2] Eva Blomberg[1], Inger Odnevall[1,3,4,]*

[1]KTH Royal Institute of Technology, Department of Chemistry, Division of Surface and Corrosion Science, SE-100 44 Stockholm, Sweden

[2]Uppsala University, Department of Chemistry – Ångström Laboratory, Box 523, 751 20 Uppsala, Sweden

[3]AIMES–Center for the Advancement of Integrated Medical and Engineering Sciences at Karolinska Institute and KTH Royal Institute of Technology, SE-100 44 Stockholm, Sweden

[4]Karolinska Institute, Department of Neuroscience, SE-171 77 Stockholm, Sweden

*Correspondence: ingero@kth.se (I.O.)

Table of contents:

Supporting Information

Page S2: Atomic absorption spectroscopy – Figure S1, Released amount of copper per particle mass.

Page S3: UV-Vis – the Ghormley triiodide method – Figure S2 and S3, H_2O_2 calibration curves from measurements with Cu NPs.

Page S5: Electron paramagnetic spin resonance – Figure S4. An example of EasySpin spectral fitting on the spectrum Figure 5C-12 min.

Atomic absorption spectroscopy

Samples containing 0 or 100 μg/ml Cu NPs and 0, 0.0025, or 5 mM H_2O_2 were mixed at room temperature and centrifuged, after 1 hour of exposure, at 50000 rpm for 1-hour rpm (Beckman Optima L-90K Ultracentrifuge, 1998). The supernatant (3.5 ml supernatant to a total volume of 10 ml) was then digested with 0.49 M H_2O_2, 0.07 M HNO_3, and MQ-water for 35 min using UV light (705 UV Digester, Metrohm). Cu concentration was determined using AAS and flame. Calibration standards were prepared from the Cu metal standard from PerkinElmer. Samples were compared to controls made from concentrated aqua regia and the Cu NPs stock solution. The limit of detection was calculated as three times the standard deviation of the background samples.

Release of Cu in PBS. The sample released Cu concentration compared to the total amount of added Cu NPs. They were analyzed with AAS and flame. Exposure 1h in PBS at 20°C. The

limit of detection was calculated to 0.005 ppm by multiplying the standard deviation of the blank samples with 3

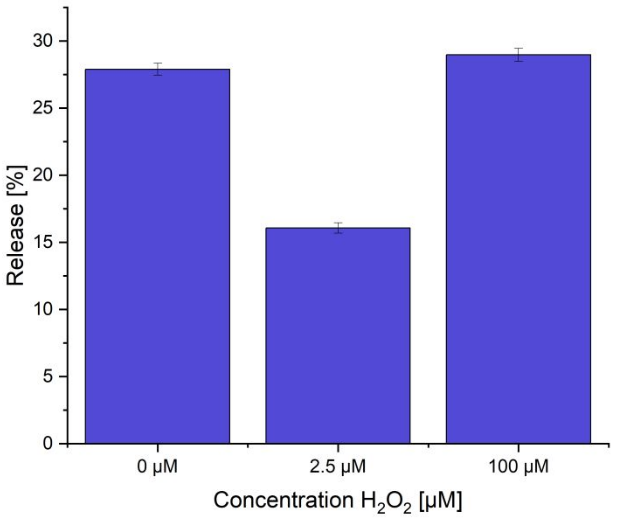

Figure S1. Released amount of copper per particle mass after 1 h of exposure in PBS with and without H_2O_2

Interestingly, there is a lower metal release with 2.5 μM H_2O_2 present compared to no H_2O_2 and a higher concentration of 100 μM H_2O_2, see Figure 4. This is likely due to passivation at a low concentration of H_2O_2, limiting the corrosion rate and thus decreasing metal release. It has previously been seen that H_2O_2 passivates copper due to oxide formation[1].

The release study revealed a decrease in Cu release in samples with an H_2O_2 concentration of 25μM, compared to 0 and 100 μM. The result indicates passivation of the Cu surface around 25

μM of H_2O_2. H_2O_2 is produced in our bodies as a part of our immune system. The biologically produced H_2O_2 could, at specific concentrations, improve the passivation of the Cu NPs and decrease the harmful effects. Outside of this passivating H_2O_2 concentration range, the corrosion rate would increase, which this study has revealed means a higher production rate of ROS.

(1) Du, T.; Vijayakumar, A.; Desai, V. (2004) Effect of hydrogen peroxide on oxidation of copper in CMP slurries containing glycine and Cu ions. *Electrochim. Acta 49* (25), 4505-4512. DOI: https://doi.org/10.1016/j.electacta.2004.05.008.

Supporting Information

UV-Vis – the Ghormley triiodide method

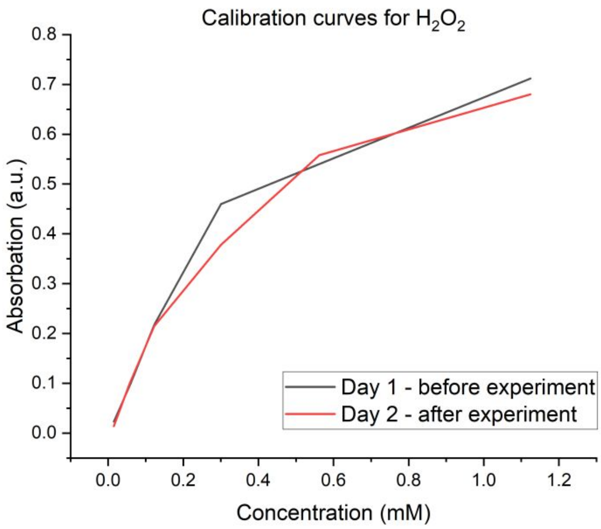

Figure S2. H_2O_2 calibration curves from measurements with 100 µg/mL Cu NPs

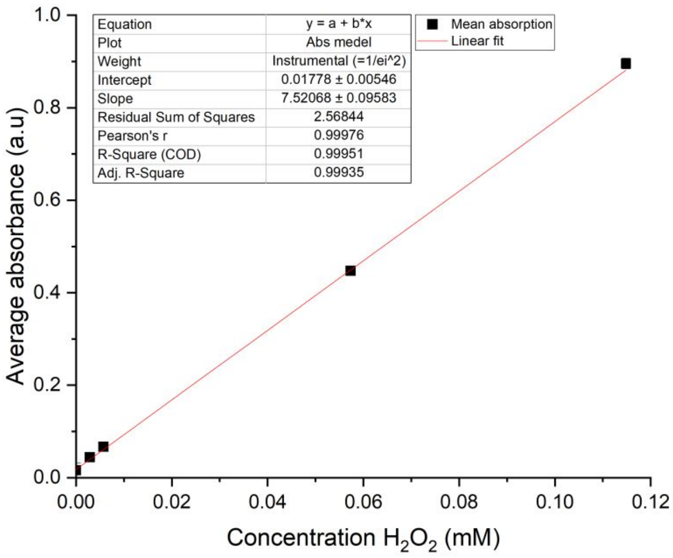

Figure S3. H_2O_2 calibration curve for measurements with 40 µg/mL Cu NPs.

Electron paramagnetic spin resonance

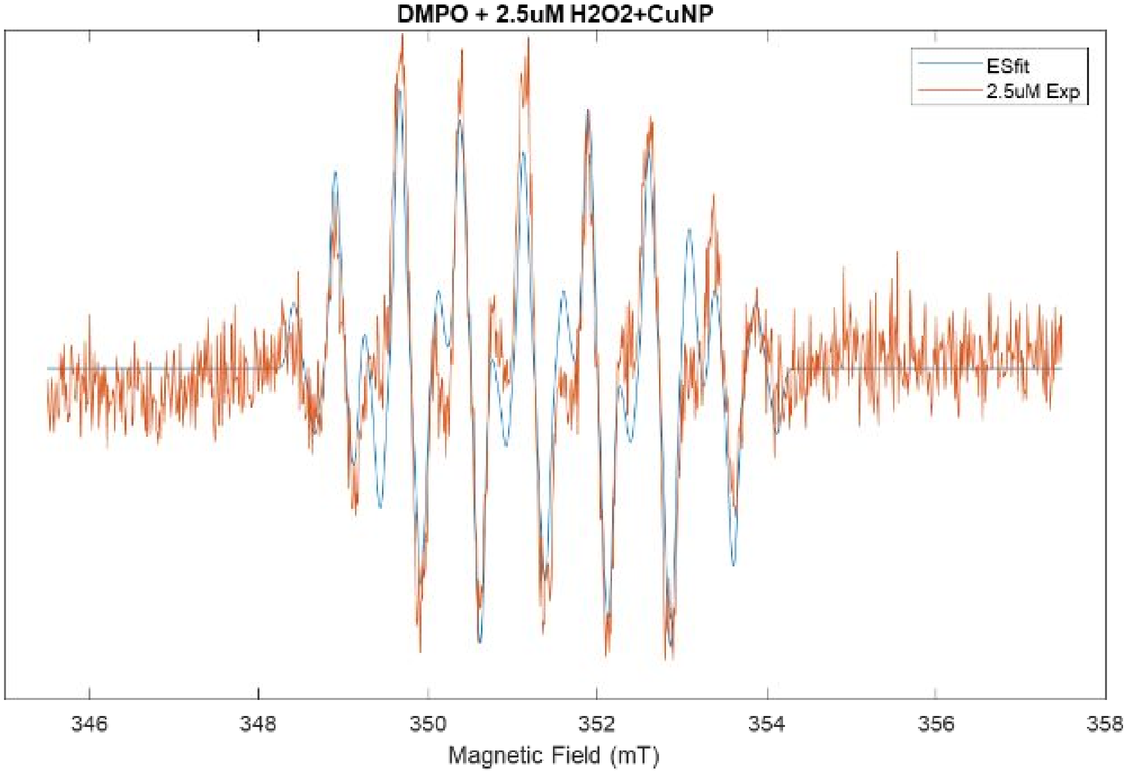

Figure S4. An example of EasySpin spectral fitting on the spectrum Figure 5C-12 min, where three components of spin-adduct were identified.
